# Upregulation of multiple key molecules is correlated with poor prognosis and immune infiltrates in hepatocellular carcinoma by bulk and single-cell RNA-seq

**DOI:** 10.18632/aging.206151

**Published:** 2024-11-12

**Authors:** Xutong Li, Jiaying Liu, Linyan Zhao, Hao Gu, Yan He

**Affiliations:** 1 Department of Immunology, School of Basic Medical Sciences, Anhui Medical University, Hefei, China; 2 Department of Infectious Diseases, Xingtai People’s Hospital, Xingtai, China; 3 Department of Gastroenterology, Nanyang Second General Hospital, Nanyang, China; 4 Department of General Surgery, The First Affiliated Hospital of Anhui Medical University, Hefei, China

**Keywords:** immune infiltration, tumor microenvironment, immunotherapy, hepatocellular carcinoma, prognostic signature

## Abstract

Background: Recent discoveries in hepatocellular carcinoma (HCC) unveil key molecules. However, due to liver cancer’s high heterogeneity, predicting patient prognosis is challenging. This study aims to construct a model for predicting HCC prognosis using multiple key genes.

Methods: TCGA provided RNA expression and clinical data, differentially analyzed by DESeq2, edgeR, and Limma. The hub gene was pinpointed via CytoHubba’s degree algorithm in Cytoscape. GO and KEGG analyses illuminated potential pathways. Single-cell sequencing detailed key gene expression in diverse cell types. The LASSO regression model predicted patient prognosis.

Result: In the RNA-seq analysis using three R packages, we identified 762 differentially expressed genes, with Cytoscape revealing ten key genes showing significant prognostic value (*P* < 0.05). GO and KEGG analyses highlighted key biological processes and pathways. IHC confirmed higher expression in cancer tissues. Reduced immune cell infiltration was observed in HCC tissues, and immune checkpoint analysis showed a strong correlation between PD1, CTLA4, and hub genes. Single-cell sequencing indicated higher expression of key genes in immune cells than hepatocytes. Cox analysis validated the riskScore as a reliable, independent prognostic marker (HR = 4.498, 95% CI: 2.526–8.007).

Conclusions: The results from differential analysis using three R packages are robust, revealing genes closely linked to immune cell infiltration in the tumor microenvironment. Additionally, a validated prognostic model for liver cancer was established based on key genes.

## INTRODUCTION

Hepatocellular carcinoma (HCC) stands as one of the prevailing malignancies globally, ranking second among causes of cancer-related mortality [1, 2]. The worldwide incidence of liver cancer approximates to 9.3 cases per 100,000 person-years, accompanied by a mortality rate of 8.5 [3]. Alarming statistics reveal a dismal five-year survival rate of less than 10% [4]. There are an estimated 841,000 new cases and 782,000 deaths each year [5]. Notably, HCC constitutes the predominant subtype, accounting for 75% to 85% of primary hepatic carcinomas [6]. Chronic infections such as hepatitis B virus (HBV) and hepatitis C virus (HCV), alongside cirrhosis and alcoholic liver disease, primarily underpin the etiology of HCC [7]. Given the multifaceted nature of its origins, therapeutic responses to HCC exhibit considerable variability. Consequently, the imperative for novel biomarkers and prognostic models in facilitating precise individualized management strategies is underscored.

In recent years, immunotherapy has revolutionized cancer treatment paradigms, representing a beacon of hope for patients worldwide [8, 9]. Immunotherapy, represented by immune checkpoint inhibitors (anti-PD-1/L1 antibody and anti-CTLA-4 antibody), has excellent efficacy in some patients. Unfortunately, immune checkpoint inhibitors (ICIs) are ineffective in most patients, and their clinical use remains limited [10–12]. This also indicates that tumors have inherent resistance to immune checkpoint blockade [13]. Immune checkpoint blockade (ICB) is often performed by enhancing immune cell infiltration in the tumor microenvironment. Comprising a dynamic milieu of immune cells, stromal components, and secreted factors, the TME orchestrates a delicate balance between tumor progression and immune surveillance [14–16]. There is increasing evidence that immune cell infiltration in the tumor microenvironment is crucial in immunotherapy [17]. While the significance of immune cell infiltration within the TME in dictating treatment outcomes is increasingly recognized, current research predominantly focuses on isolated molecular or cellular markers. Consequently, there remains a notable gap in our understanding of the collective impact of multiple key genes and immune infiltration patterns on immunotherapy response [18, 19].

The purpose of this study was to evaluate the efficacy of immunotherapy by integrating several key genes and observing their relationship with immune cell infiltration and their correlation with immune checkpoints.

## MATERIALS AND METHODS

### Data source

We downloaded transcriptome data and clinical data about HCC from the TCGA cohort from the UCSC Xena website (https://xena.ucsc.edu/), including 374 liver hepatocellular carcinoma (LIHC) samples and 50 normal samples [20]. The clinical data included survival time, survival status, sex, age, TNM stage and grade. In addition, corresponding mutation data were downloaded from the TCGA database for subsequent mutation analysis [21].

### Identification of differentially expressed genes (DEGs)

RNA expression profiles of HCC and normal samples were obtained from the database. The RNA sequencing data of HCC included more than 50,000 genes. To ensure the accuracy of differential analysis, we used three existing R packages including DESeq2, edgeR and Limma [22–24]. We obtained differentially expressed genes (DEGs) using three analysis methods, and the significant DEGs were selected with the cutoff criteria *P*-value < 0.05 and |logFC|≥ 2. We obtained 1997 genes from DESeq2 differential analysis, 2146 genes from edgeR differential analysis and 1564 genes from Limma differential analysis. A total of 762 intersected genes were obtained from the intersection of the three gene sets.

### Functional annotation and gene enrichment analysis

To explore and obtain the potential biological processes and signaling pathways of differential genes, the clusterProfiler R package was used to perform gene ontology (GO) and KEGG enrichment analysis [25, 26]. The GO enrichment analysis covers biological process (BP), molecular functions (MF) and cellular components (CC). In addition, GSEA was performed on the high- and low-risk group of the follow-up prognostic model [27]. The annotated gene set file is “c2.cp.kegg.v7.4.symbols.gmt”. Determined the threshold as NOM *p*-value < 0.05.

### Analysis of the PPI network and hub genes

The genes obtained by differential analysis were then analyzed by Kaplan–Meier (KM) analysis to obtain indexes related to survival. We submitted these genes to the STRING database (https://cn.string-db.org/) to establish a network diagram of interactions between proteins. Then, the Degree algorithm of the Cytohubba plug-in of Cytoscape software was used to analyze the differential genes, and 10 hub genes were obtained. Then, we used the maftools package for mutation analysis of the 10 genes [28].

### Immunohistochemical staining

The Human Protein Atlas (HPA) database (https://www.proteinatlas.org/) contains the immunohistochemical results of various tissues and corresponding cancers. We found immunohistochemical staining of proteins, including AURKA, CCNB1, CDC20 and TOP2A, in liver cancer tissues and normal tissues.

### Immunoassay

In the study of the expression matrix, we used the ssGSEA method to quantify the abundance of cell infiltrates of various immune cells in each sample, as described in Charoentong et al. [29]. A total of 28 human TME cell subtypes were evaluated, including Activated B cell, Activated CD4 T cell, Activated CD8 T cell, Central memory CD4 T cell, Central memory CD8 T cell, Effector memory CD4 T cell, Effector memory CD8 T cell, Gamma delta T cell, Immature B cell, Memory B cell, Regulatory T cell, T follicular helper cell, Type 1 T helper cell, Type 17 T helper cell, Type 2 T helper cell, Activated dendritic cell, CD56^bright^ natural killer cell, CD56^dim^ natural killer cell, Eosinophil, Immature dendritic cell, Macrophage, Mast cell, MDSC, Monocyte, Natural killer cell, Natural killer T cell, Neutrophil, and Plasmacytoid dendritic cell. To assess differences in the immune microenvironment between normal and tumor tissues, we derived the immune cell score, stromal cell score, and total score using an ESTIMATE algorithm [30]. We also calculated the correlation between hub genes and immune checkpoints and DNA repair genes through Spearman correlation analysis to judge whether hub genes are suitable for predicting the efficacy of immunotherapy [31, 32].

### Single-cell data analysis

Single-cell sequencing data were obtained from the GSE146115 dataset (https://www.ncbi.nlm.nih.gov/), comprising samples from four cases of liver cancer. We processed the data and conducted analysis using the Seurat package, followed by dimensionality reduction through PCA and T-SNE clustering. The SingleR package was utilized for cell type annotation in the single-cell data, analyzing the composition of various cell types within tumors and assessing the expression of pivotal genes across different cell types.

### Prognostic model construction

Based on the integrated role of 10 key genes in liver cancer progression, we constructed a riskScore model to comprehensively evaluate the role of these molecules in patient prognosis. The prognostic model was established by the least absolute shrinkage and selection operator (LASSO) Cox regression analysis [33]. The penalty parameter (λ) for the model was determined by tenfold cross-validation following the minimum criteria. The number of related genes was determined by the λ value. The riskScore of each HCC patient was calculated by the formula: riskScore = (Expression level of Gene 1 × coefficient) + (Expression level of Gene 2 × coefficient) + … + (Expression level of Gene *n* × coefficient). The surv_cutpoint function was used to determine the optimal truncation value, and the samples were divided into high and low expression groups. The prognostic model could be verified by survival analysis. ROC curve was used to analyze the efficacy of this prognostic model. The riskScore can be used to explore the correlation with immunization.

### Statistical analysis

The Wilcoxon test was used to analyze the difference between the two groups. The correlation analysis between the two sets of data is based on the Spearman correlation test. The Kaplan-Meier method and log-rank test were used to estimate OS. Cox regression analysis was performed via the R package “survival”, along with hazard ratios (HRs) and 95% confidence intervals (CIs). All *P*-values were bilateral, and *P* < 0.05 was considered statistically significant. R Software (Version 4.1.2) was used to perform statistical analysis and plotting.

### Data availability statement

The raw data supporting the conclusions of this article will be made available by the authors, without undue reservation. Article/Supplementary Materials include the original contributions presented in the study. Please contact the corresponding authors for further information. The following is a link to the raw data: https://xena.ucsc.edu/, https://www.proteinatlas.org/, https://www.ncbi.nlm.nih.gov/. Immunohistochemical images of different genes can be found at: https://www.proteinatlas.org/ENSG00000087586-AURKA, https://www.proteinatlas.org/ENSG00000134057-CCNB1, https://www.proteinatlas.org/ENSG00000117399-CDC20, https://www.proteinatlas.org/ENSG00000131747-TOP2A.

## RESULTS

### Differential genes between normal and liver cancer tissues

**Figure 1:**
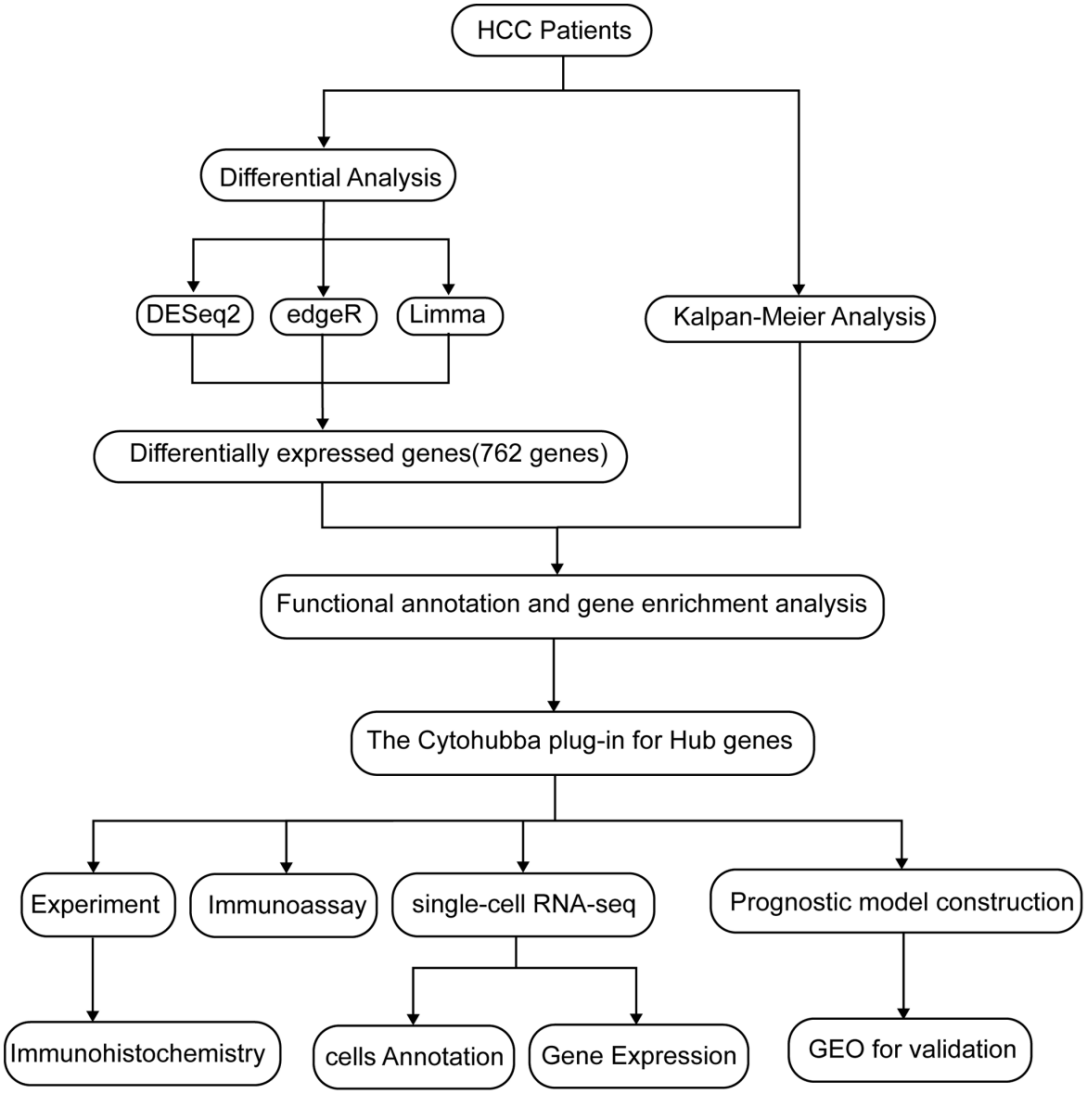
A flow chart of the manuscript.

First of all, we draw a flow chart, so that readers can better understand the context of the article (Figure 1). RNA-seq data and corresponding clinical data of liver cancer were obtained from the TCGA database, and genes with extremely low expression values were eliminated. In addition, to ensure the reliability of the difference analysis results, we conducted difference analysis on the data through the DESeq2, edgeR and limma R packages. DESeq2 analysis showed that 1997 genes were significantly different between tumor and normal samples (*P* < 0.05 and | logFC |≥ 2), while EdgeR and Limma analyzed 2146 and 1564 genes, respectively. Hierarchical clustering clearly shows the genomic differences between normal and tumor tissues with three difference analysis methods (Supplementary Figure 1A). In addition, in order to more intuitively display the number of up-regulated and down-regulated genes in the differential analysis, we drew a volcano map. We can see that 1719 up-regulated genes and 278 down-regulated genes were detected with DESeq2 package, while 1882 up-regulated genes and 264 down-regulated genes were detected with edgeR package. Finally, 512 genes were up-regulated and 1052 down-regulated when analyzed with Limma package (Supplementary Figure 1B).

### Functional enrichment analysis

**Figure 2:**
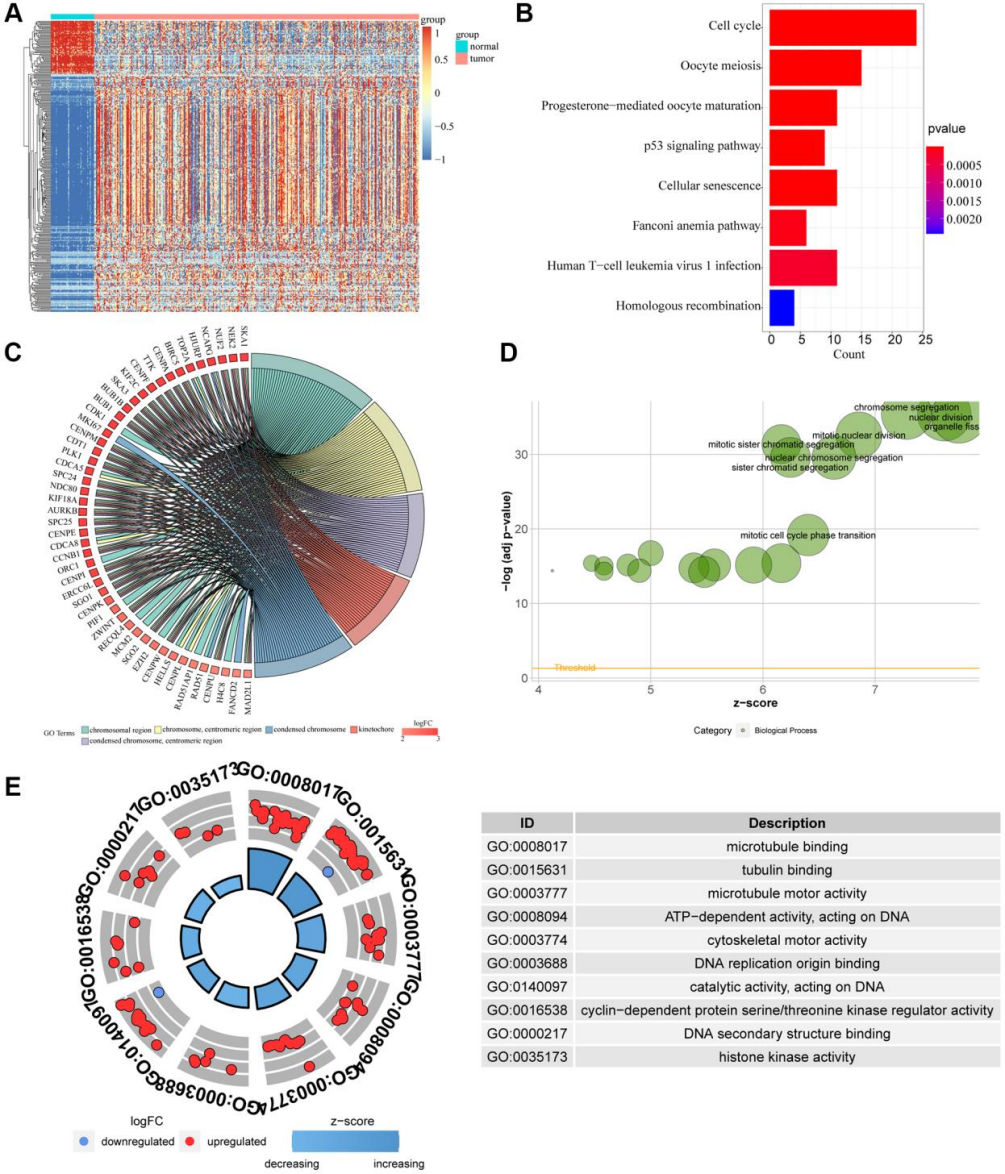
**Difference of genomic landscape between normal and LIHC tissues.** (**A**) Hierarchical clustering visualizing the intersections of DEGs with KM analysis. (**B**–**D**) Gene Ontology functional enrichment analyses for differentially expressed genes. (**B**) Biological process. (**C**) Molecular function. (**D**) Cellular component. (**E**) KEGG pathway enrichment analyses for differentially expressed genes. All enriched pathways were significant. The color depth represented enriched adjusted *p*-value.

**Figure 3:**
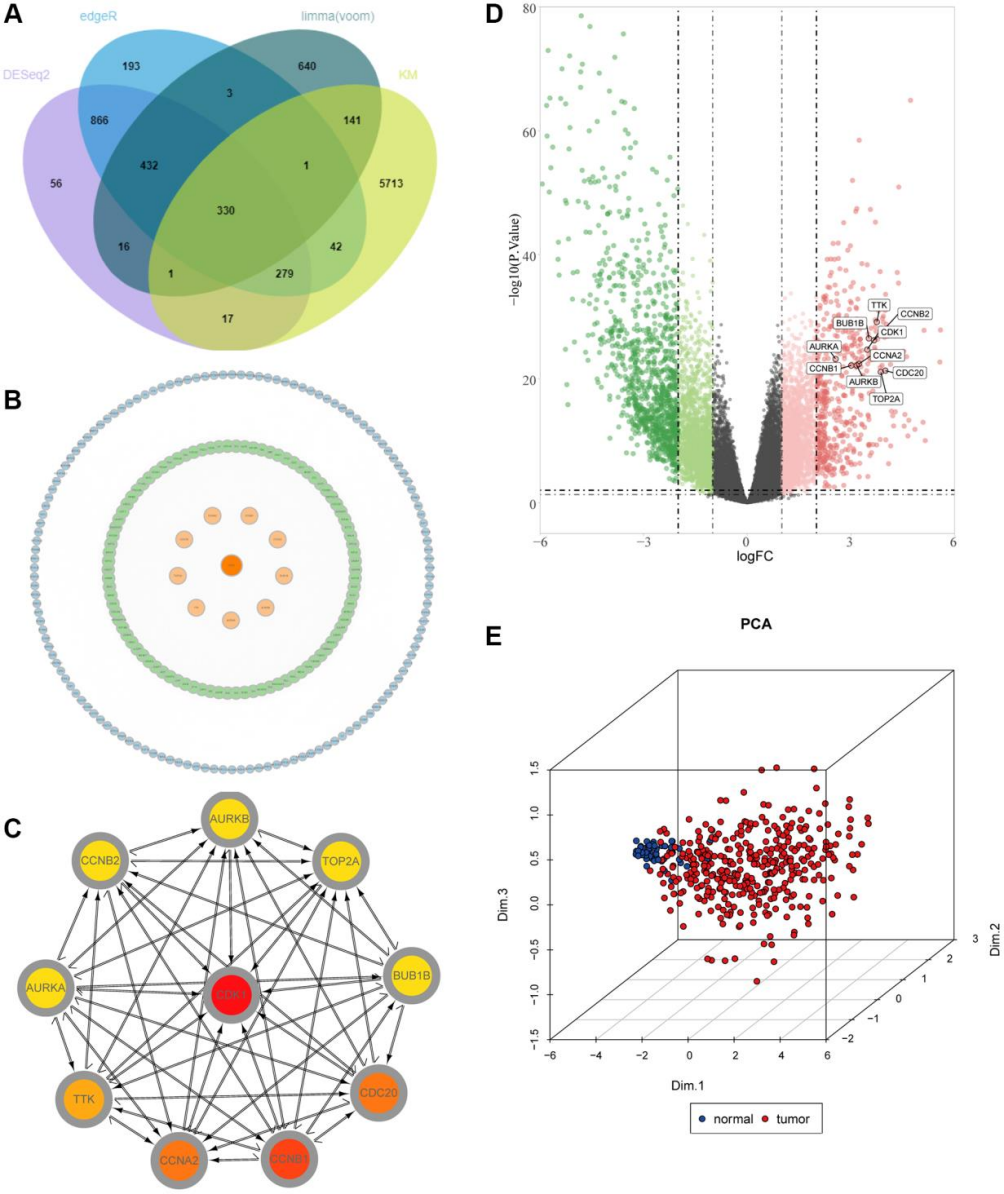
**Identification of key molecules in LIHC.** (**A**) Venn diagram visualizing the intersections of DEGs with KM analysis. (**B**) Construction of a protein–protein interaction (PPI) network among differentially expressed genes. (**C**) The relationship among the ten key molecules at the protein level. (**D**) Volcano plot constructed with the cut-off criterion *p* < 0.05 and |logFC|≥ 1. Red, up-regulated genes; Green, down-regulated genes. The circle represented each gene and the identified key molecules were marked. (**E**) Principal component analysis for the key molecules revealed two completely disjoint populations, suggesting these key molecules could well distinguish LIHC samples from normal samples. Blue, normal samples; Red, tumor samples.

We obtained 1997 genes from DESeq2 differential analysis, 2146 genes from edgeR differential analysis and 1564 genes from Limma differential analysis. A total of 762 intersected genes were obtained from the intersection of the three gene sets. We used heat maps to show the expression of 762 differential genes obtained by 3 differential analyses in cancer tissues and adjacent tissues (Figure 2A). KM survival analysis of these genes showed that 330 genes were closely related to survival (*P* < 0.05) (Figure 3A). Then, we performed GO and KEGG analysis on these genes. When we performed BP analysis, it was mainly enriched to chromosome segregation, while CC was mainly enriched to chromosomal region, which fully indicated that these genes were closely related to chromosomes (Figure 2C, 2D). In addition, Molecular Function analysis revealed that ATP-dependent activity, DNA replication, catalytic activity acting on DNA, and DNA secondary structure binding were predominantly enriched. These MF fully demonstrate that these genes are intimately associated with DNA replication (Figure 2E). Interestingly, KEGG analysis found that these genes are also rich in the cell cycle, cellular senescence and other signaling pathways related to the cell cycle. The cell cycle is often closely related to the occurrence and development of tumors (Figure 2B).

### PPI network and hub gene analysis

We constructed PPI networks for the 330 genes mentioned above (Figure 3B). First, the interaction network between proteins was searched through the STRING database, and then 10 hub genes were screened out by the degree algorithm in the CytoHubba plug-in, including CDK1, CCNB1, CCNA2, CDC20, TTK, TOP2A, AURKA, AURKB, BUB1B and CCNB2 (Figure 3C). In addition, we mapped the volcanoes of these ten genes (Figure 3D). We performed PCA clustering analysis on the samples and found that the samples could be clustered into two categories (Figure 3E). Then, we conducted a difference analysis and survival analysis on the 10 genes, and we found that all 10 genes were meaningful (*P* < 0.05) (Supplementary Figure 2A, 2B). Meanwhile, immunohistochemical results of AURKA, CCNB1, CDC20 and TOP2A were found through the HPA database, which also confirmed that there were significant differences between liver cancer tissues and normal adjacent tissues (Supplementary Figure 3A). Correlation analysis found that all 10 genes were positively correlated with each other (Supplementary Figure 3B). Through mutation analysis conducted by maftools, we found that the mutation types were mainly missense mutations (Supplementary Figure 3C).

### Immunoassay

**Figure 4:**
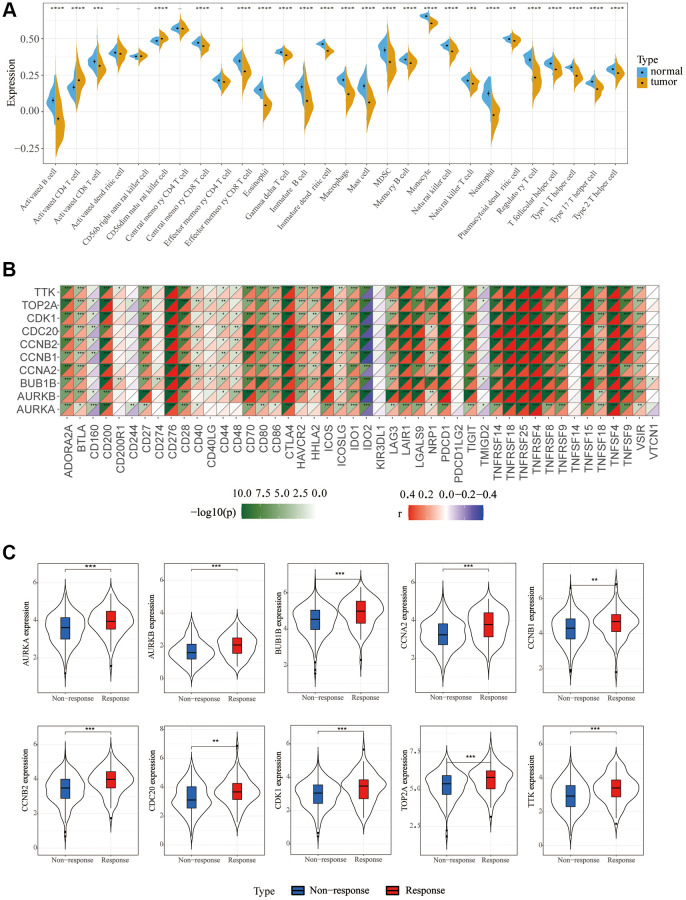
**Immune cell infiltration and correlation analysis.** (**A**) Differences in 28 TME infiltration cells between normal liver and LIHC tissues (^*^P < 0.05; ^**^P < 0.01; ^***^P < 0.001). (**B**) The correlation between each key molecule and each immune checkpoint. Red, positive; Purple, negative. (**C**) Immunotherapy efficacy of 10 key genes.

**Figure 5:**
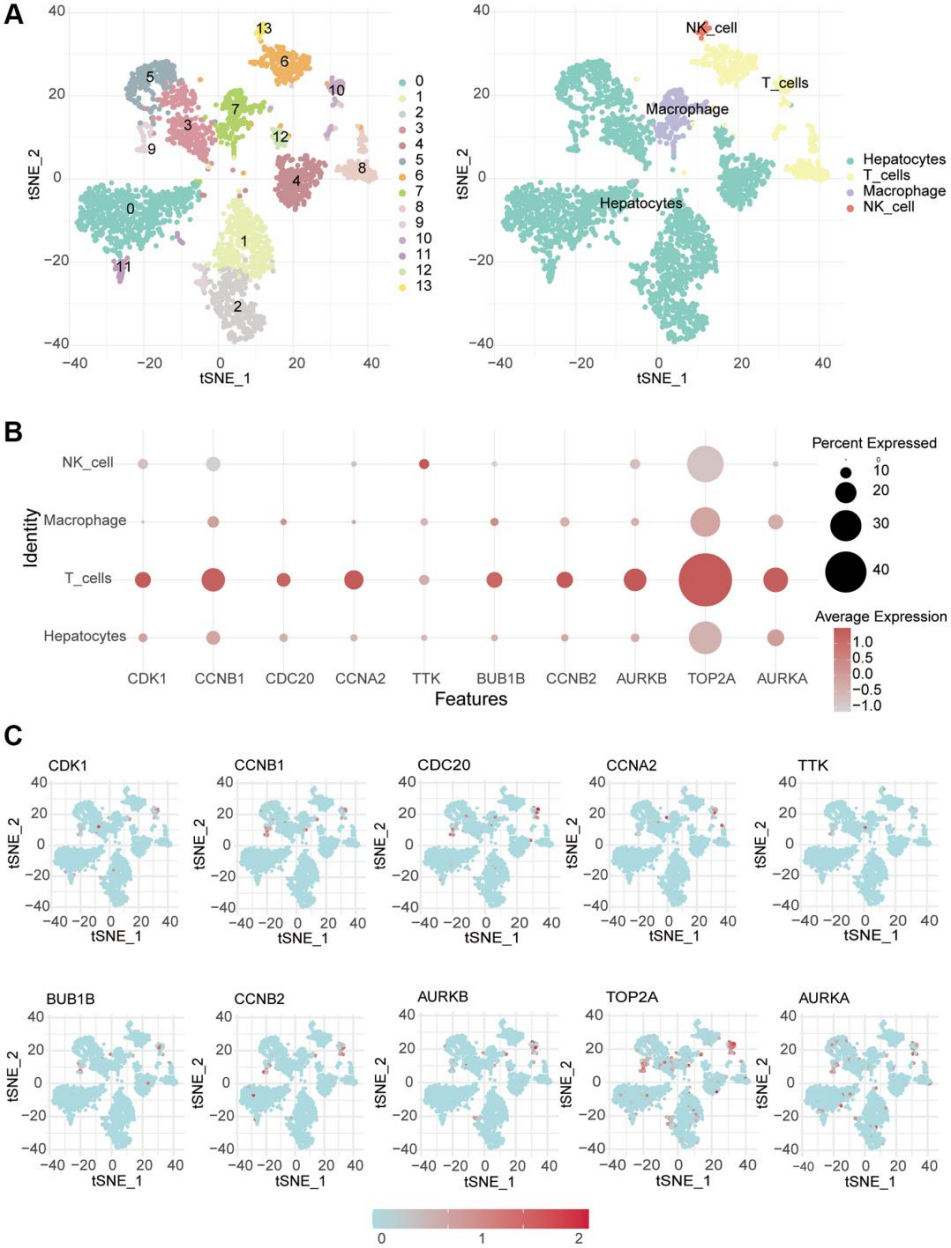
**Single-cell RNA-seq analysis.** (**A**) The different cells were annotated. (**B**, **C**) Single-cell analysis was used to monitor the expression of 10 key genes in different immune cells.

To explore the relationship between screened hub genes and the tumor microenvironment, we calculated the immune abundance of each sample according to Charoentong’s article and the ssGSEA algorithm. We found that except for activated CD4 T cells and CD56^dim^ natural killer cells, the expression level in cancer tissues was higher than that in normal tissues, and the expression level of most other immune cells was low in cancer (Figure 4A). Through the TIMER2.0 (http://timer.cistrome.org/) database, we further examined the correlation between these molecules and immune cell subtypes and found that the results were basically consistent with ours (Supplementary Figure 4). We used the Estimate algorithm to find that immune and stromal scores were higher in normal tissue than in tumor tissue (Supplementary Figure 5A). Then, correlation analysis between immune checkpoints and hub genes was carried out, and it was found that hub genes were positively correlated with multiple immune checkpoints, including PD1 and CTLA4, but PDL2 was not associated with hub genes (Figure 4B). For DNA repair genes, the correlation between MSH2, MSH6 and hub genes was very high (Supplementary Figure 5B). In addition, we found that these key genes respond well to immunotherapy (Figure 4C). Single-cell sequencing data were used to analyze the differences in cellular components in the tumor microenvironment. It can be seen that in addition to hepatocytes, macrophages, T cells and NK cells also have higher expression levels (Figure 5A). The expression of hub genes in different cells was then analyzed and it was found that most of the hub genes were mainly expressed in T cells. Among them, TOP2A showed the highest expression in various cells compared with other hub genes (Figure 5B, 5C).

### Prognostic model construction

**Figure 6:**
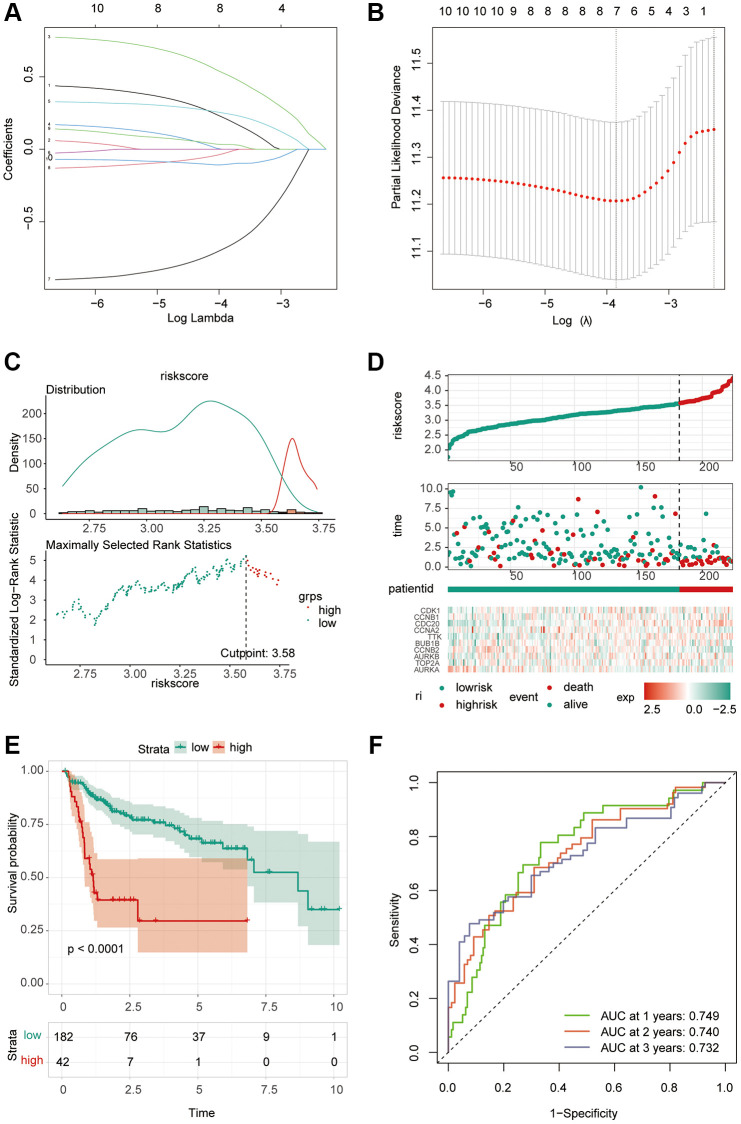
**Construction of riskScore signature.** (**A**) Least absolute shrinkage and selection operator (LASSO) coefficient profiles of the ten key molecules. (**B**) Penalty plot for the LASSO model for the 10 prognostic genes with error bars denoting the standard errors. (**C**) The optimal cut-off point to dichotomize riskScore into low and high groups was determined by the function surv_cutpoint. The optimal cut-off point was 3.58. (**D**) Proportion of deaths in high and low risk groups as riskScore values increased. Hierarchical clustering of seven key genes between low and high risk groups. Red, up-regulated; Blue, down-regulated. (**E**) Survival analyses for low and high riskScore groups using Kaplan-Meier curves. (*P* < 0.0001, Log-rank test) (**F**) Predictive efficacy of riskScore on prognosis.

**Figure 7:**
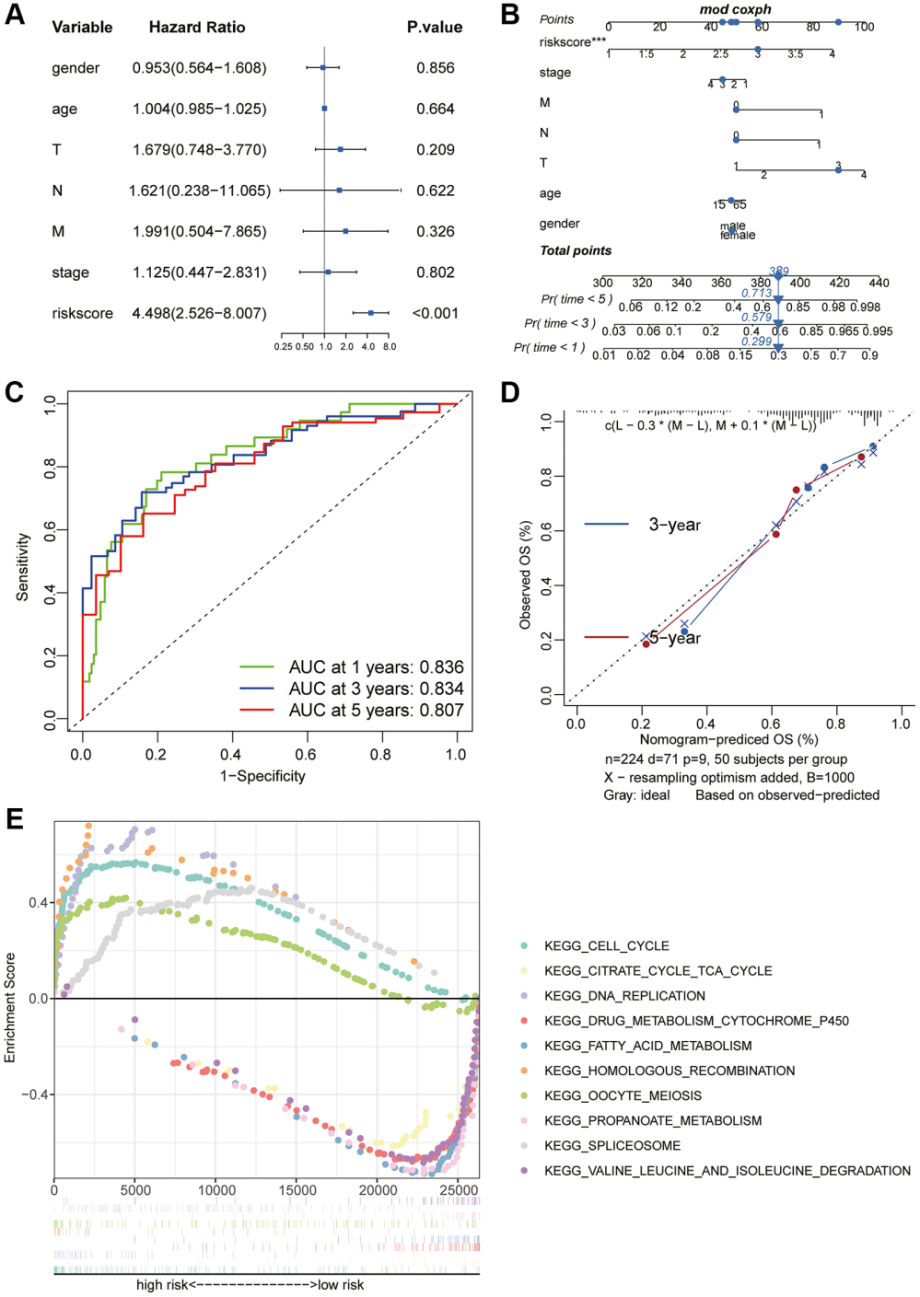
**Prognostic value of the riskScore gene signature.** (**A**) Forest plot showing the riskScore was an independent prognostic biomarker using multivariate analyses. (**B**) The nomogram was constructed to predict the probability of patient mortality. (**C)** The Predictive efficacy of nomogram score on prognosis. (**D**) The calibration plot of nomograms between predicted and observed 3-year and 5-year outcomes. The 45-degree line represented the ideal prediction. (**E**) The GSEA enrichment reveals two significantly activated signaling pathways, including the cell cycle pathway.

Considering the correlation between these key molecules and patient prognosis, we modeled the hub gene. We used Lasso Cox regression analysis to construct the model with 4 genes, including CDC20, TTK, CCNB2 and AURKA, and calculated the riskScore for each sample (Figure 6A, 6B). We then used the “surv_cutpoint” function to determine that the optimal cutoff value for the riskScore was 3.58 (Figure 6C). According to the riskScore, patients were divided into the high- and low-risk group for survival analysis, and it was found that the low-risk group had a significant survival benefit (Figure 6D, 6E). The ROC curve analysis found that the model had better predictions (Figure 6F). Univariate Cox regression analysis included patient age, sex, clinical grade, and TNM stage (Figure 7A). The results showed that the riskScore could be used as a robust and independent prognostic biomarker to evaluate the prognosis of HCC patients. To establish a method to quantitatively predict patient outcomes in combination with clinical outcomes, we established a nomogram risk map combining riskScore and clinical factors (Figure 7B). At the same time, we calculated the nomogram score. The ROC curves were evaluated by the scores, and we found excellent prediction results (Figure 7C). The calibration diagram shows that the derived line map performs well compared to the ideal model (Figure 7D). To explore the biological pathways between the high-risk and low-risk groups, we also performed GSEA and found that the cell cycle was significantly activated in the high-risk group (NOM *p*-value = 0.01) (Figure 7E).

### Risk score immunoassay and model verification

**Figure 8:**
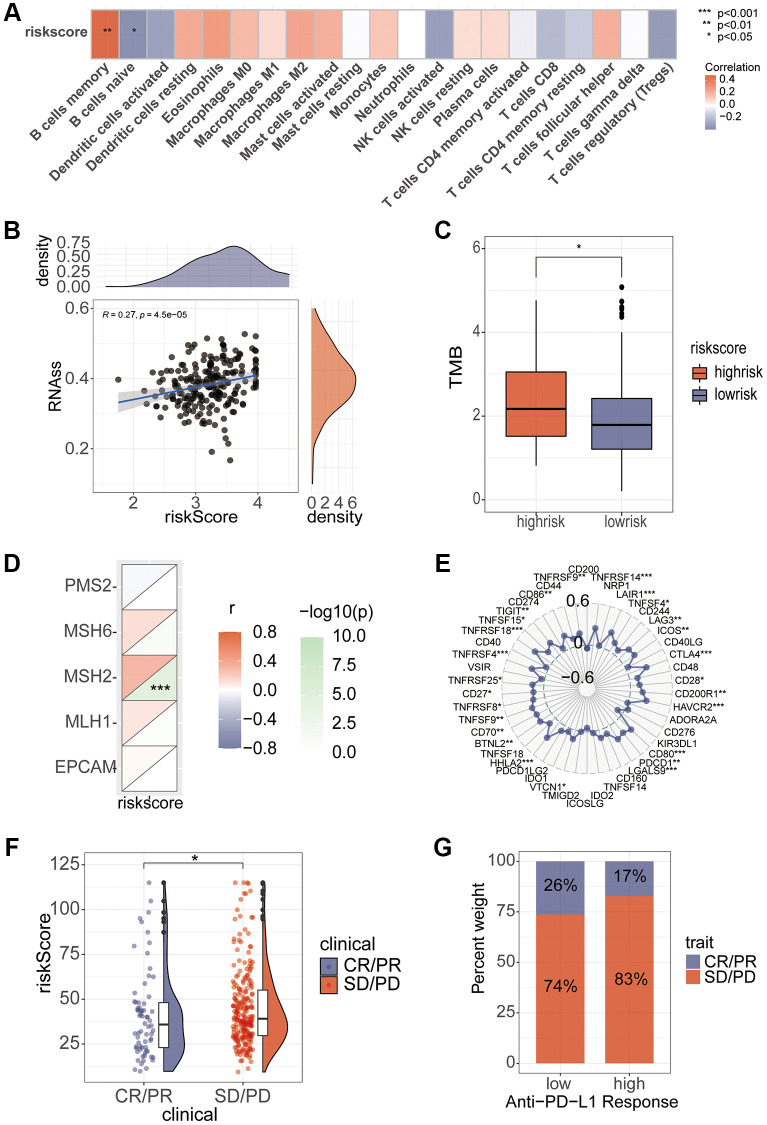
**Association of riskScore with immunity.** (**A**) Correlation of riskScore with immune cells. (**B**) Tumor stem cell relevance. (**C**) Differences in TMB scores in high and low risk groups. (**D**, **E**) Correlation of Mismatch Repair gene and immune checkpoints with riskScore. (**F**, **G**) Assessing differences in risk scores between immune efficacy groups.

**Figure 9:**
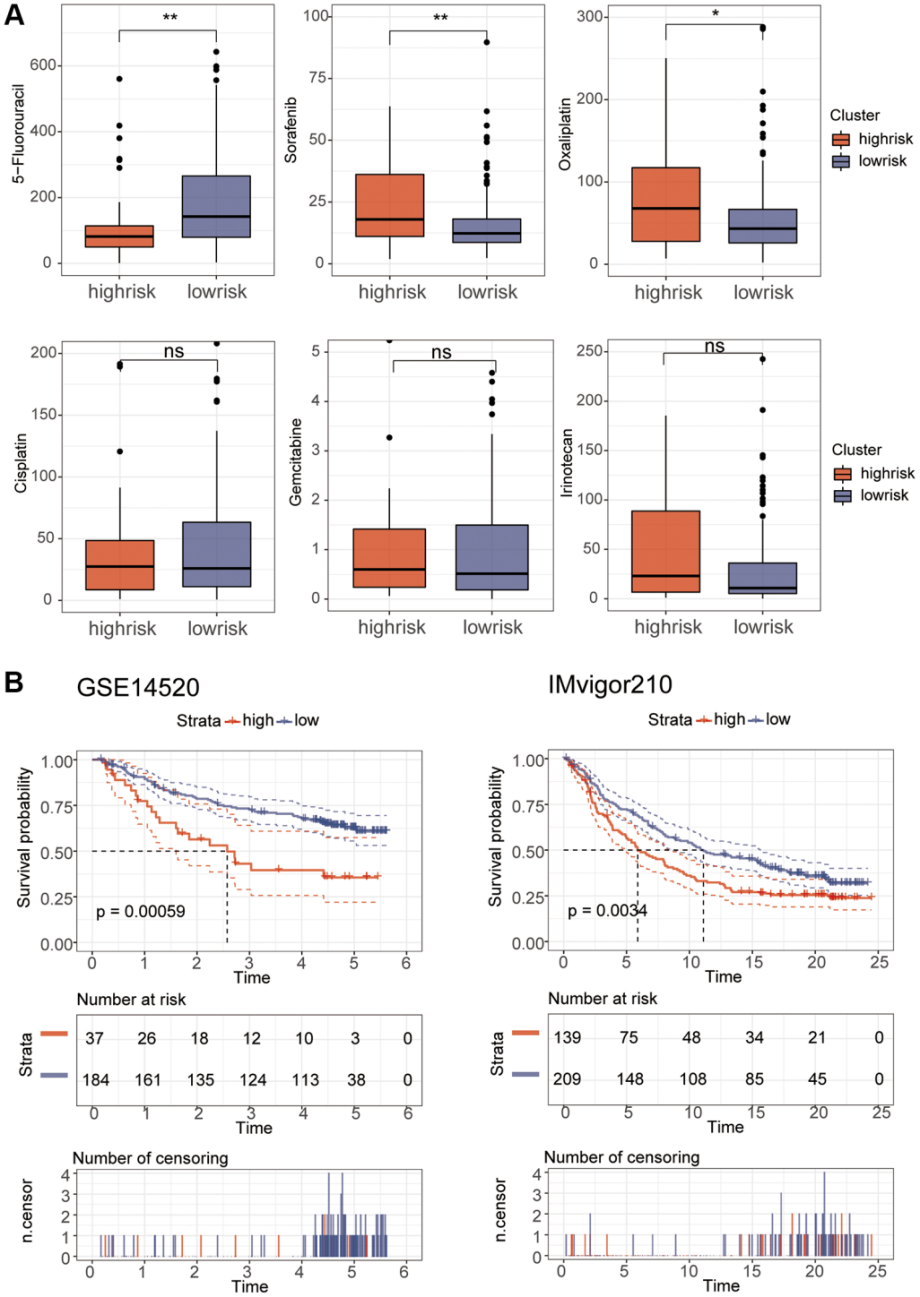
**Drug sensitivity and model validation.** (**A**) The sensitivity of various drugs was assessed between the high and low risk groups. (**B**) GSE14520 and IMvigor210 were used to verify the accuracy of the model.

Correlation analysis found that B cell memory and B cell naive had a high correlation with the riskScore (Figure 8A). In addition, stem cell correlation analysis revealed a significant correlation between them (Figure 8B). We analyzed the relationship between TMB and riskScore, and TMB score was significantly higher in the high-risk group than in the low-risk group (Figure 8C), and the correlation analysis of MMR and immune checkpoints also found a high correlation between risk scores and several indicators (Figure 8D, 8E). Immunotherapy analysis using the IMvigor210 package showed significant differences in immune efficacy between high and low risk groups (Figure 8F, 8G). Differential analysis of the high and low risk groups for various different chemotherapeutic agents revealed that Sorafenib, 5-Fluorouracil, and Oxaliplatin differed between the two groups (Figure 9A). Subsequent validation of the model using GSE14520 and IMvigor210 revealed that patients in the high-risk group had a significantly worse prognosis than those in the low-risk group (Figure 9B).

## DISCUSSION

At present, the treatment of liver cancer is still mainly surgery, but for patients in the middle and late stages, the recurrence rate of tumors is very high [34]. With the advent of tumor immunotherapy, immune checkpoint inhibitors have been used as one of the new effective methods for tumor therapy [11]. However, only 17–18% of patients with advanced HCC respond to anti-PD1 antibody therapy [2]. Therefore, it is difficult to know whether immunotherapy is effective in a particular patient, and how to screen out those patients who respond is a clinical problem that needs to be solved.

Based on TCGA transcriptome and clinical data, three R packages were used for differential analysis. Then, we intersected the results and found 762 genes. Survival analysis of these genes revealed that 330 genes were significant. Previous studies often used one of the three R packages of DESeq2, edgeR and Limma for differential analysis, and the results inevitably had some errors. However, we selected the intersection of genes for differential analysis through the three R packages to ensure the reliability of differential genes. In addition, the cell cycle was enriched in signaling pathways. According to the research of Suski et al., the CELL cycle is found in almost all cancers and is one of the causes of cancer occurrence [35]. In addition, Tuo et al. found that PCK1 can lead to the occurrence of HCC by targeting the cell cycle [36]. These findings also confirm our results. Then, we calculated 10 hub genes through the degree algorithm in Cytohubba, and they were highly expressed in tumor samples. This is proved by HPA database. Those with low gene expression had better survival. Yin et al. found that siRNA knockdown of CDK1, CCNB1 and CCNB2 could significantly induce autophagy and senescence of HCC cells [37]. CDC20 is a WD40 activator for a cell cycle degradation machine [38]. CCNA2, TTK, TOP2A, AURKA, AURKB and BUB1B are also closely associated with cancer [39–41]. By immunoassay, we found that these genes were closely related to multiple immune cells. Meanwhile, the expression abundance of immune cells was significantly reduced in HCC, and the estimation algorithm also found that the immune cell score and stromal cell score in the immune microenvironment were significantly decreased in HCC samples. There is increasing evidence that some key proteins are crucial molecules in the immune microenvironment and can regulate the tumor microenvironment. And these 10 key genes are closely associated with immunotherapy. Single-cell data analysis found that hub genes were mainly expressed in T cells in the tumor microenvironment. In addition, we established a risk model for 10 genes through Lasso-Cox regression analysis, and we found that the model had good predictive value. Similarly, Qiong et al. also predicted the prognosis of liver cancer well by establishing a prediction model [42]. Additionally, we used the model to assess its correlation with the immune system, revealing a close relationship between the model’s riskScore and B cells. Studies have shown that B cells are crucial regulatory factors in the hepatocellular carcinoma microenvironment and are closely associated with the development and progression of HCC [43]. Furthermore, we found that the risk score was closely correlated with MSH2, PD1, and CTLA4, indicating that this risk score could effectively predict the immunotherapy response in patients, which is of significant guidance for immunotherapy. Further analysis of immunotherapy revealed disease remission in the low-risk group, further confirming the close correlation between the risk score and immunotherapy. Interestingly, GSEA showed that the cell cycle was mainly enriched in the high-risk group, which was consistent with the KEGG results above, indicating that these genes were closely related to the cell cycle.

This study has important clinical application value. We found that differential analysis with three R packages was more reliable, and the hub gene helped to screen suitable patients for immune checkpoint inhibitor therapy. Research on the correlation between hub genes and MMR is helpful in judging the effect of immunotherapy. Furthermore, the constructed riskScore signature can be used as a reliable and independent biomarker to predict the prognosis of HCC patients. Targeting these 10 key molecules, which are closely related to immune cell infiltration, will contribute to the development of personalized tumor immunotherapy.

There are several limitations to our study that need to be acknowledged. First, the study was an analysis using a public database and lacked validation of our own cohort. We will further study these hub genes in our own HCC data cohort. Second, the downstream targeted genes of these 10 genes have not been further explored, which may lead to certain deviations in the estimation of targeted drugs, which also requires further research. Third, the LIHC transcriptome analysis used to construct riskScore is based on the Illumina RNA-seq platform. Therefore, we should be cautious when applying the riskScore signature to LIHC samples tested using other platforms.

## CONCLUSIONS

The genes obtained by the difference analysis of three R packages were more reliable. We found that the hub gene was closely related to immune cell infiltration and played a huge role in immunotherapy. In addition, these genes can well predict the prognosis of liver cancer by constructing models.

## Supplementary Materials

Supplementary Figures
