# Supplementary material for: Upregulation of multiple key molecules is correlated with poor prognosis and immune infiltrates in hepatocellular carcinoma by bulk and single-cell RNA-seq
Source: Aging (Albany NY). 2024 Nov 12;16(21):13371–91. doi: 10.18632/aging.206151 (PMC11719102; doi:10.18632/aging.206151)
Supplement: Supplementary Figures [file aging-16-206151-s001.pdf]

SUPPLEMENTARY FIGURES

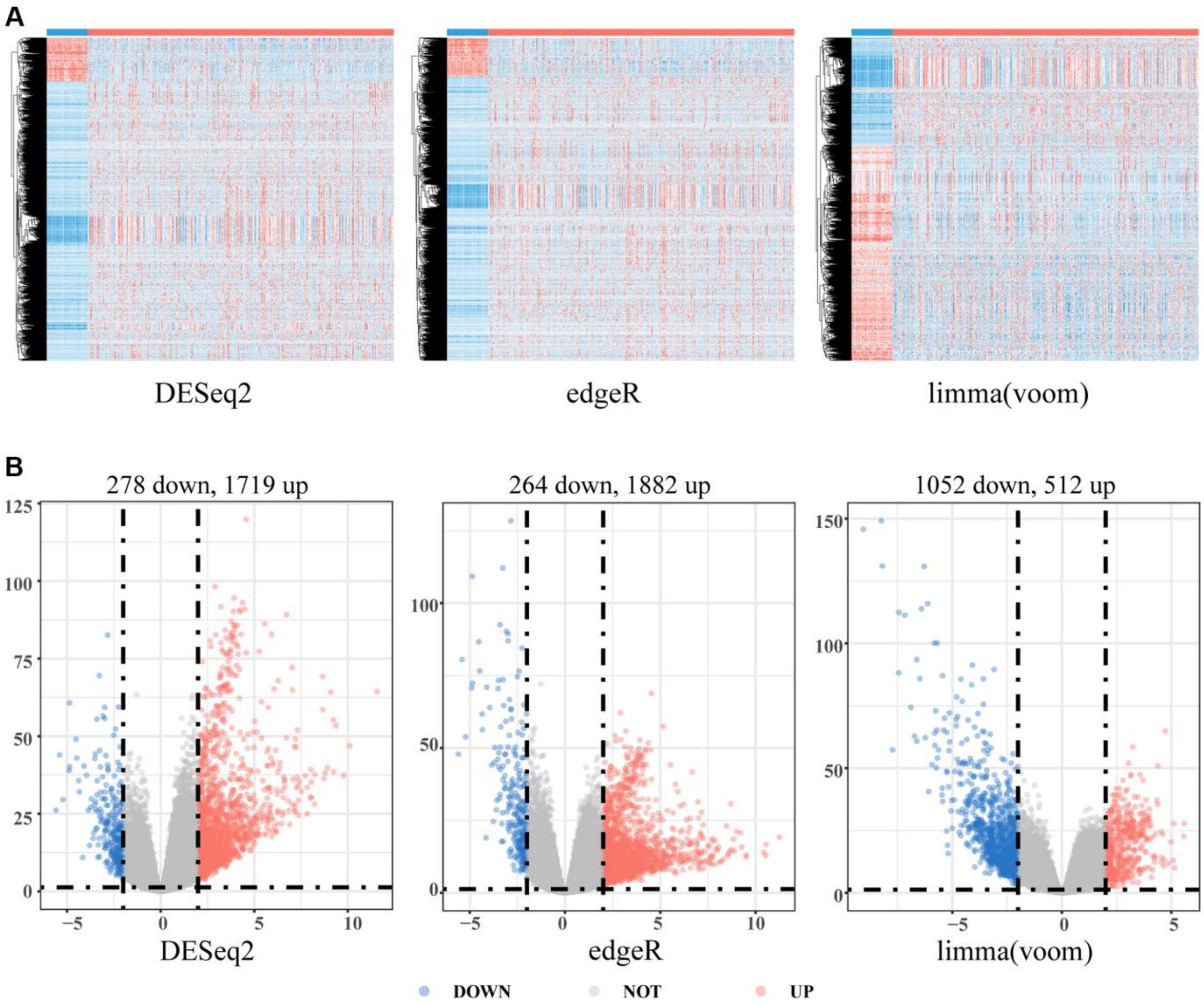

Supplementary Figure 1. Three R packages difference analysis. (**A**) The difference analysis heat map of R packages including DESeq2, edgeR and limma. (**B**) The difference analysis volcano map of R packages including DESeq2, edgeR and limma.

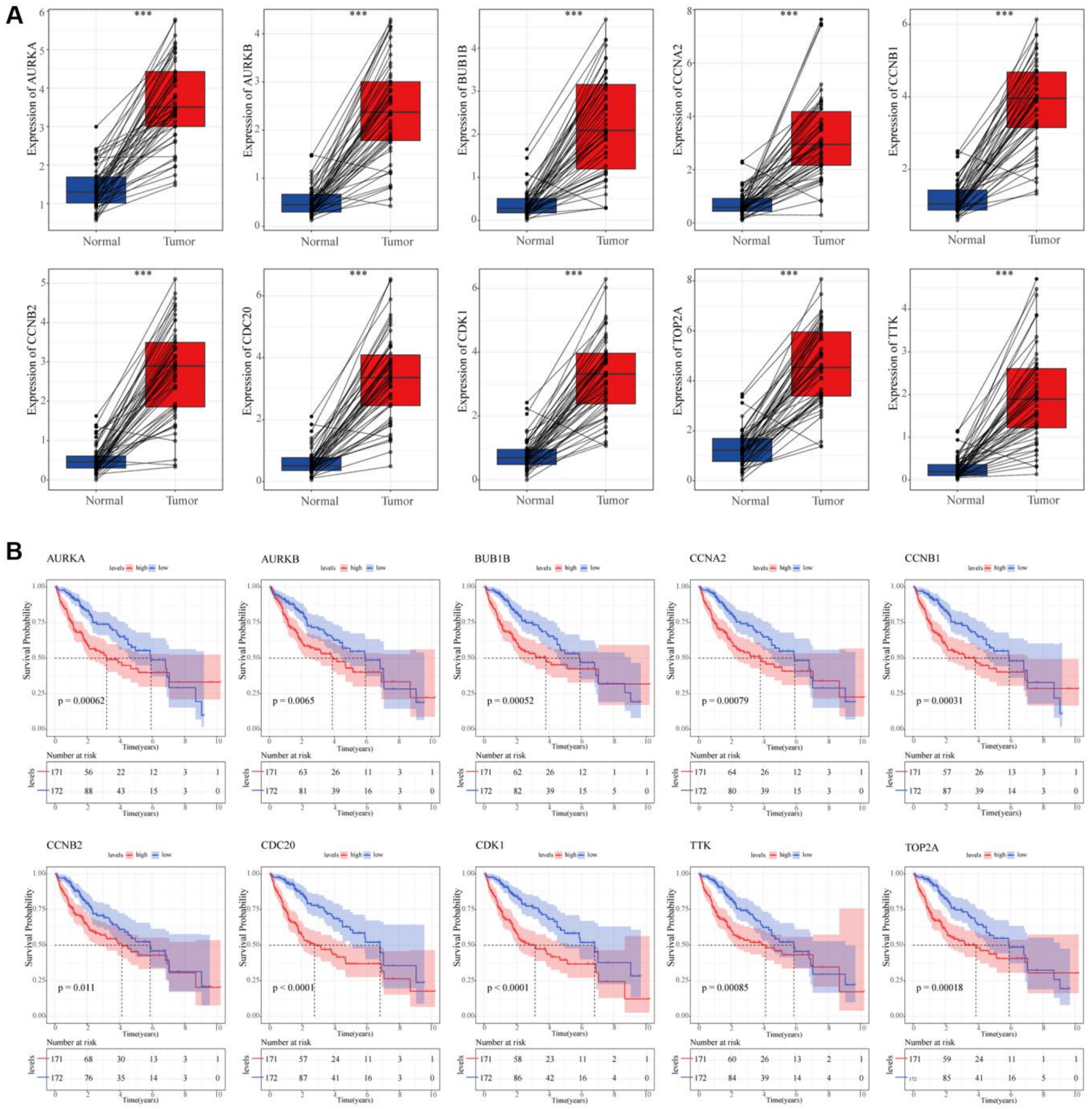

Supplementary Figure 2. Expression analyses and survival analyses for the ten key molecules. (**A**) Differential analysis of key genes. (**B**) Survival analysis of key genes.

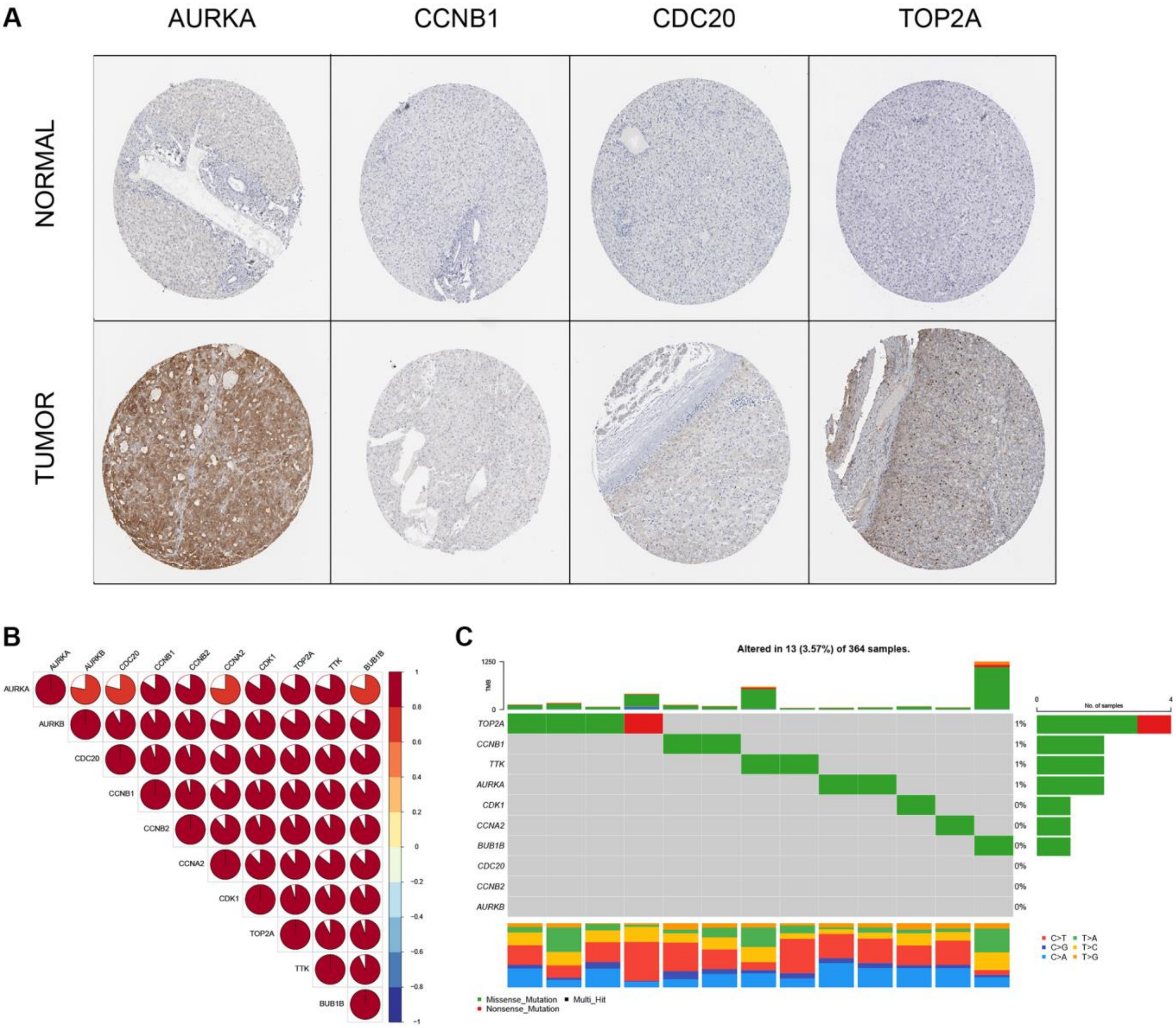

Supplementary Figure 3. Multi-omics analyses of identified key molecules. (**A**) Immunohistochemical results of HPA database. (**B**) The ten key genes were positively correlated. (**C**) Mutation analysis of 10 genes.

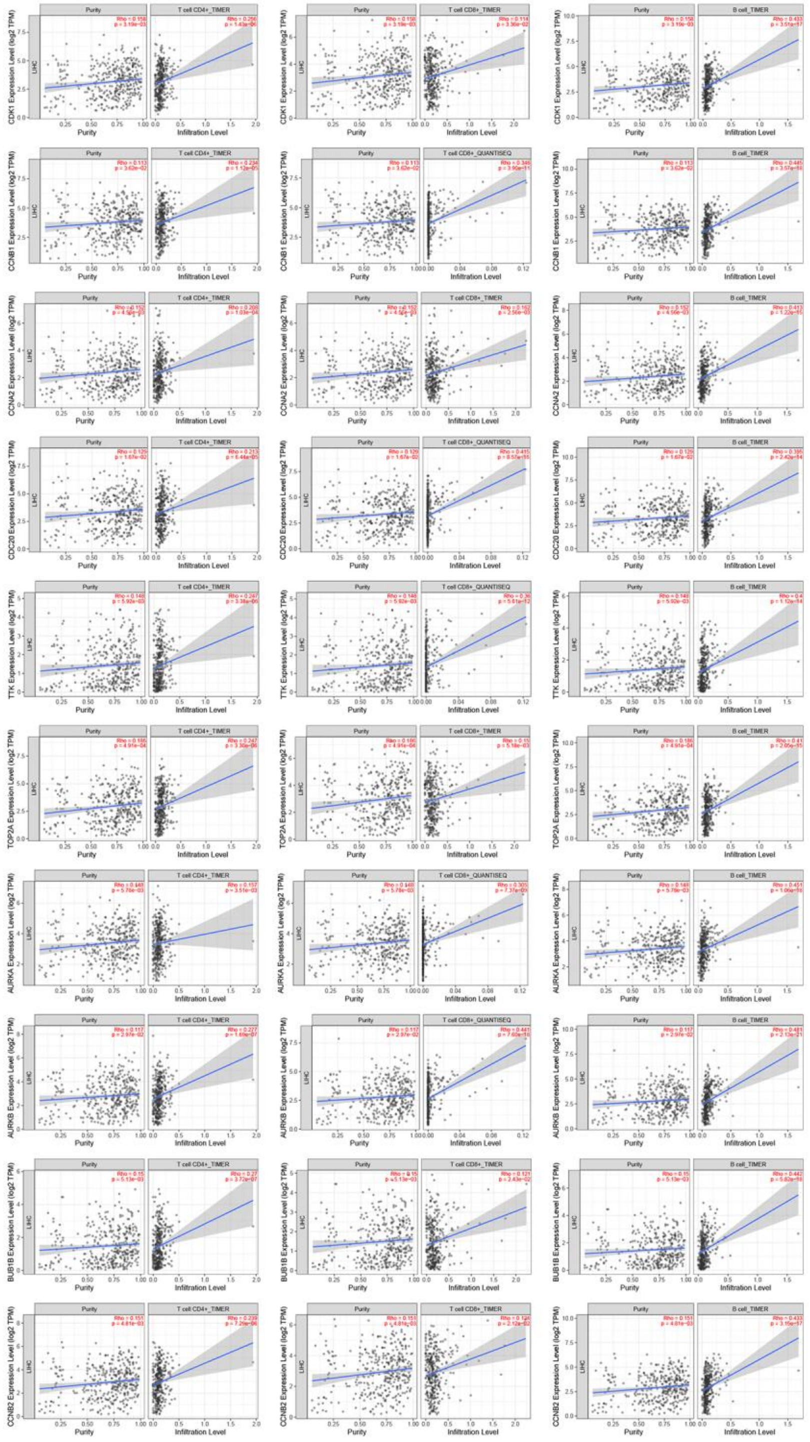

Supplementary Figure 4. The correlation between 10 key genes and immune cells was shown in the Timer database. The correlation between 10 key genes and immune cells was shown in the Timer database.

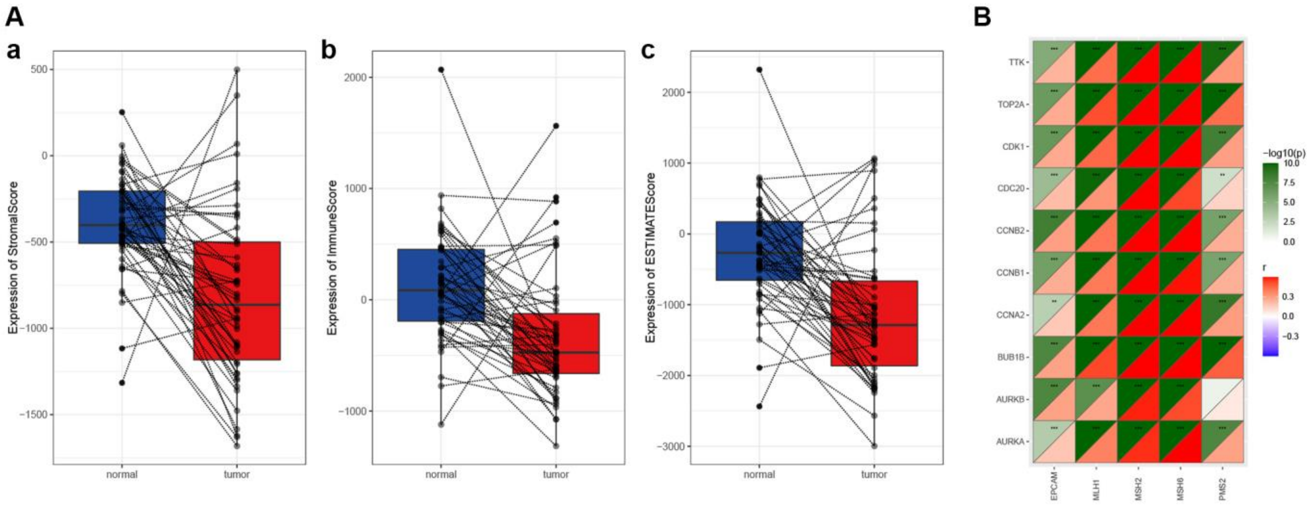

Supplementary Figure 5. The tumor microenvironment and DNA repair gene analysis. (**A**) The immune and matrix scores in the tumor microenvironment were lower in HCC than in normal tissues. (**B**) Key genes are closely related to multiple DNA repair genes.
